# Supplementary material for: An open-source framework for large-scale, flexible evaluation of biomedical text mining systems
Source: J Biomed Discov Collab. 2008 Jan 29;3:1. doi: 10.1186/1747-5333-3-1 (PMC2276192; doi:10.1186/1747-5333-3-1)
Supplement: Additional file 1 — Evaluation platform source code. The additional file contains the software evaluation framework discussed in this paper. The most current release can be downloaded from SourceForge.net [1]. See the accompanying README file for instructions on installation and use. Also included in the distribution is a collection of UIMA wrappers for some commonly used BioNLP tools and annotated corpora. [file 1747-5333-3-1-S1.zip › bionlp_uima_repository-1.0.1/docs/api/src-html/edu/uchsc/ccp/util/nlp/annotation/printer/SimpleCollectionReaderFormatOutput.html]

```
001    package edu.uchsc.ccp.util.nlp.annotation.printer;
002    
003    import java.io.PrintStream;
004    import java.util.List;
005    
006    import edu.uchsc.ccp.util.nlp.document.GenericDocument;
007    
008    /**
009     * THis class outputs the document text and documentid into a format that can be read in by the SimpleCollectionReader, namely documentID|documentText
010     * @author williamb
011     *
012     */
013    public class SimpleCollectionReaderFormatOutput implements AnnotationPrinterUtil {
014        private boolean useOneLineFormat = false;
015        
016        public void printDocumentCollection(List<GenericDocument> documents) {
017            printDocumentCollection(documents, System.out);
018        }
019        
020        public void printDocumentCollection(List<GenericDocument> documents, PrintStream ps) {
021            for (GenericDocument gd : documents) {
022                printDocument(gd, ps);
023            }
024        }
025        
026        public void printDocument(GenericDocument gd) {
027            printDocument(gd, System.out);
028        }
029        
030        public void setUseOneLineFormatStatus(boolean useOneLineFormat) {
031            this.useOneLineFormat = useOneLineFormat;
032        }
033        
034        public void printDocument(GenericDocument gd, PrintStream ps) {
035            String documentID = gd.getDocumentID();
036            String documentText  = gd.getDocumentText();
037            
038            ps.println(documentID + "|" + documentText);
039        }
040        
041        
042    }
```
